# Consensus Molecules Associated with Parkinson’s Disease

**DOI:** 10.3390/neurolint18020023

**Published:** 2026-01-27

**Authors:** Sara Eyal, Shira Alfasi, Karin Ben Zaken, Ibrahim O. Sawaid, Lior Segev, Samuel Mesfin, Pnina Frankel, Rahaf Ezzy, Trishna Saha-Detroja, Shilpa Madhavan, Naamah Bloch, Baruh Polis, Abraham O. Samson

**Affiliations:** 1 Azrieli Faculty of Medicine, Bar Ilan University, Safed 1311502, Israel; 2 Hurvitz Brain Sciences Program, Sunnybrook Health Sciences Centre, University of Toronto, Toronto, ON M4N 3M5, Canada

**Keywords:** Parkinson’s disease, vitamins, cofactors, coenzyme Q10, tetrahydrobiopterin

## Abstract

Parkinson’s disease (PD) has been associated with some types of food and drugs. Here, we query PubMed for the association of PD with foods and drugs, using a list of 217,776 compounds derived from the Human Metabolome Database (HMDB). To calculate associations, a Python script was developed to query PubMed for co-citations of PD with each compound, and adjust this count for compound abundance. Notably, PD is found to be associated with small-molecule drugs, adjunctive therapies, contraindicated drugs, diagnostic agents, biomarkers, conditional essential molecules, and inducers. Drugs include L-dopa (49%), carbidopa (63%), benserazide (50%), entacapone (74%), tolcapone (56%), rasagiline (76%), selegiline (46%), pargyline (4%), ropinirole (61%), pramipexole (56%), lisuride (27%), cabergoline (16%), bromocriptine (12%), and zonisamide (9%). Adjunctive therapies include droxidopa (33%), trihexyphenidyl (28%), biperiden (17%), amantadine (24%), memantine (7%), rivastigmine (13%), donepezil (6%), galantamine (4%), domperidone (6%), clonazepam (4%), tetrabenazine (16%), mazindol (13%), quetiapine (6%), and clozapine (4%). Contraindicated drugs include haloperidol (4%), sulpiride (3%), and methyldopa (6%). Diagnostic agents include FP-CIT (60%) and beta-CIT (43%). Biomarkers include 3-methoxytyrosine (48%) and homovanillic acid (12%). Endogenous cofactors include tetrahydrobiopterin (4%) and Coenzyme Q10 (4%). Chemical inducers of PD include 6-hydroxydopamine (40%), N-Methyl-4-phenyl-1,2,3,6-tetrahydropyridine (MPTP, 78%), tetrahydropyridine (77%), probenecid (4%), quinolinic acid (4%), 1,2,3,4-tetrahydroisoquinoline (TIQ, 16%), salsolinol (32%), rotenone (25%), and β-Methylamino-L-alanine (BMAA, 29%). Notably, our study highlights conditional essential endogenous cofactors associated with PD and emphasizes rational directions for investigation in PD.

## 1. Introduction

Parkinson’s disease (PD) is a neurodegenerative disorder that afflicts the dopaminergic system of the central nervous system (CNS) [1]. Symptoms include tremors, rigidity, bradykinesia, gait disturbances, insomnia, and cognitive impairments. Its gradual progression is marked by characteristic atrophy of the substantia nigra, which produces dopamine [2]. PD characteristics include the buildup of α-synuclein that disrupts mitochondrial homeostasis [3]. The aggregation of α-synuclein is associated with iron accumulation and oxidative stress [4]. Furthermore, mitochondrial dysfunction promotes ferroptosis [5]. PD etiology is not completely understood, and some of the risk factors are believed to be inherited, involving single or multiple genes [6]. Other risk factors include oxidative stress [7], mitochondrial dysfunction [8], viral infections [9,10], diabetes [11], head trauma [12], low body mass index (BMI) [13], exposure to harmful chemicals, and environmental factors. The common denominator of these risk factors is that they all contribute to cellular stress, particularly by disrupting redox balance and perturbing mitochondrial function.

Current treatments for PD include selective therapies, such as dopamine precursors (e.g., levodopa), aromatic L-amino acid decarboxylase (AADC) inhibitors (e.g., carbidopa), catechol-O-methyltransferase (COMT) inhibitors (e.g., entacapone), and dopamine receptor agonists (e.g., bromocriptine). These selective therapies are often preferred over non-selective treatments like monoamine oxidase B (MAOB) inhibitors (e.g., selegiline) [14]. Notably, MAOB inhibitors suffer from a well-known drug interaction with tyramine, and tyramine-rich foods can lead to a dangerous pressor reaction and hypertensive crisis [15]. Therefore, MAOB inhibitors should not be taken with tyramine-rich nutrients such as red wine and aged cheese (e.g., Stilton). Finally, L-dopa is best taken 1–2 h before meals containing proteins, as protein-rich diets impair its absorption [16]. While these therapies provide symptomatic relief, they do not halt neurodegeneration and often lead to long-term complications such as dyskinesia and psychiatric side effects.

Nutrients play a key role in PD, and certain foods and drugs contribute to its development. For example, dietary fat intake affects PD risk, although this depends on the fatty acid subtype [17]. While a high intake of polyunsaturated fatty acids (PUFAs) reduces the risk of PD, excessive consumption of arachidonic acid and cholesterol increases it [18]. Notably, omega-3 fatty acids have been linked with neuroprotective effects [19]. Alcohol consumption has also been linked to PD, though studies with a higher risk of selection bias found only a weak correlation [20]. Metal ions have also been linked to PD, and while excessive metal levels in the brain are harmful, deficiency negatively impacts brain function [21]. Notably, low levels of copper, iron, zinc, and lead, as well as high levels of these metals, have been associated with PD cases [22]. Low levels of polyphenols and other antioxidants have also been associated with PD, and consumption of flavonoids and anthocyanins, which reduces the risk of PD and associated mortality [23]. Likewise, licorice, curcumin, and cocoa, which are rich in flavonoids and other polyphenols, improve motor function in PD patients [24]. Other antioxidants, such as vitamins C and E, as well as β-carotene, have been studied for their potential in reducing PD risk [25]. While vitamin E has a neuroprotective effect and attenuates the risk of PD, the studies did not suggest any protective effects associated with vitamin C or β-carotene. Additionally, vitamin D deficiency has been linked with PD, with a high frequency of deficiency noted in patients with the condition [26]. Furthermore, vitamin D deficiency correlates with disease severity and progression, but not with the age of onset. Likewise, deficiencies in B vitamins—such as B6, B12, and folic acid (B9)—have been linked with PD, and a lack thereof worsens symptoms. Importantly, vitamins B12 and folic acid play essential roles as cofactors in homocysteine metabolism, and their levels have an underlying association with PD onset and progression [27,28]. Thus, while deficiencies in vitamins B, D, and E can lead to PD, supplementation with antioxidants and phytochemicals presents an enormous potential role in the management of PD. Notably, these nutrients and vitamins occupy a unique metabolic niche in PD.

Bioinformatics uses big data to analyze biomedical data, and citation counts have often been used to identify trends and patterns in medicine [29]. In the past, we have used frequency analysis of PubMed citations and have shown that antibiotic resistance follows a cyclic pattern [30]. Furthermore, we have used PubMed frequency analysis to classify autoimmune diseases based on co-citation [31,32]. In addition, we have used citation counts to rank proton pump inhibitors with the risk of developing cancer [33]. Finally, we have used PubMed frequency analysis to find comorbid conditions with Alzheimer’s disease [34].

Currently, several million people are affected by Parkinson’s disease (PD) globally, and more than 175,000 PubMed citations are related to the disorder, thus providing a wealth of information. Here, we explore the associations between PD and nutrients. Then, we classify them according to their function and provide an overview of conditional essential molecules associated with the disease.

## 2. Results

**Figure 1:**
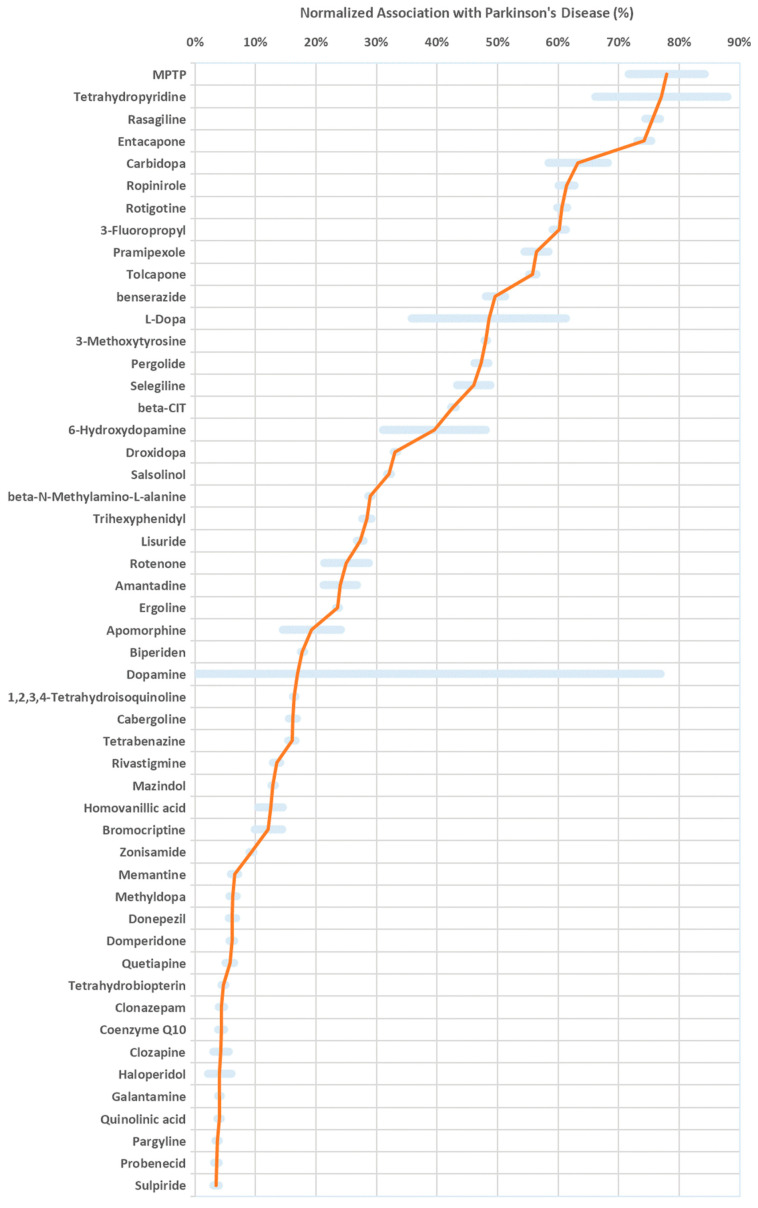
Foods and drugs associated with Parkinson’s disease (PD). Shown are the top normalized associations (percent value, red line) and co-citations (arbitrary units, blue bars) with PD according to PubMed. Associations are not indicative of causation, nor do they distinguish between positive and negative correlations.

Here, we query PubMed citations for co-citations of Parkinson’s disease (PD) with foods and drugs, using a list of 217,776 molecules from the Human Metabolome Database (HMDB 5.0) [35]. Figure 1 shows the top normalized association with PD, above an arbitrary minimum threshold of 3%, and with more than 100 co-citations. The 3% normalized association threshold was empirically selected to exclude noise while retaining biologically and clinically relevant compounds. Notably, the top associations include drugs used in the treatment of PD, adjunctive therapies, contraindicated drugs, endogenous cofactors, diagnostic markers, biomarkers, and PD inducers.

### 2.1. Drugs in the Treatment of PD

Top associations include drugs used in the treatment of PD, such as L-dopa (49%), carbidopa (63%), entacapone (74%), tolcapone (56%), rasagiline (76%), selegiline (46%), pargyline (4%), ropinirole (61%), rotigotine (61%), pramipexole (56%), pergolide (47%), lisuride (27%), apomorphine (19%), bromocriptine (12%), and zonisamide (9%). L-dopa (49%), also known as levodopa, is the precursor of dopamine (17%), and while the former can cross the blood–brain barrier (BBB) and serve as a prodrug for dopamine, the latter cannot [36]. Carbidopa (63%) and benserazide (50%), which inhibit aromatic-L-amino-acid decarboxylase (AADC), prevent L-dopa depletion outside the brain. Entacapone (74%) and tolcapone (56%), which are inhibitors of cathechol-O-methyltransferase (COMT), prevent L-dopa depletion into 3-O-methyldopa. In addition, top associations include rasagiline (76%), selegiline (46%), and pargyline (4%), which selectively and irreversibly inhibit monoamine oxidase B (MAOB) and prevent L-dopa depletion. Nevertheless, MAOB inhibitors are not the first line of defense in the treatment of PD due to potential interactions [37]. Importantly, MAOB inhibitors are safer than MAOA inhibitors, as they do not prevent the metabolism of epinephrine, norepinephrine, and serotonin, which increases the risk of a hypertensive crisis [38]. Top associations also include dopamine agonists such as ropinirole (61%) [39], rotigotine (61%) [40], pramipexole (56%) [41], pergolide (47%) [42], lisuride (27%) [43], apomorphine (19%) [44], cabergoline (16%) [45], and bromocriptine (12%) [46]. Of these, pergolide, lisuride, cabergoline, and bromocriptine are all ergoline (24%) alkaloids that were first isolated from ergot, a fungus that infects rye and causes ergotism [47]. Dopamine agonists are rarely used in PD due to the risk of valvular and lung fibrosis, and pergolide has been removed from the US market as a result. Finally, top associations also include zonisamide (9%), a sulfonamide with multiple beneficial roles in the treatment of PD. Such roles include inhibition of monoamine oxidase, blockade of T-type calcium channels, increase in the levodopa–dopamine metabolism, rise in dopamine receptor expression, as well as neuroprotection [48].

### 2.2. Adjunctive Therapy in PD

Top associations also include drugs used for the symptomatic treatment of PD, among others, to prevent falls, dyskinesia, dementia, psychosis, and dysarthria. For example, droxidopa (33%), an adrenergic agonist, has been used in patients to alleviate neurogenic orthostatic hypotension associated with PD [49]. Trihexyphenidyl (28%) and biperiden (17%), muscarinic antagonists, have been used as an antispasmodic drug to treat muscle rigidity in PD [50,51]. Amantadine (24%) and memantine (7%), inhibitors of NMDA receptors, have been used in the treatment of dementia associated with PD, as well as the treatment of motor symptoms [52,53]. Rivastigmine (13%), donepezil (6%), and galantamine (4%) are acetylcholinesterase inhibitors and have also been used in the treatment of dementia associated with PD [54,55,56].

The top associations also include domperidone (6%), a dopamine antagonist, which, minimally, crosses the BBB. Domperidone is considered the gold standard for safe treatment of gastrointestinal symptoms, such as nausea and vomiting, in patients with PD, because the risk of developing extrapyramidal adverse effects is considered minimal, yet not without potential cardiotoxicity [57]. The top associations also include clonazepam (4%). Clonazepam is a benzodiazepine that has been used in the treatment of sleep disorders associated with PD [58], as well as in the treatment of speech problems associated with PD, such as dysarthria [59]. Tetrabenazine (16%) and mazindol (13%) are tricyclic compounds that reversibly block vesicular monoamine uptake, and have been used to treat levodopa-induced dyskinesia and chorea [60], and to treat patients in the early stages of PD [61].

Associations also include quetiapine (6%) and clozapine (4%), atypical antipsychotics, used off-label for treating psychosis in PD. Quetiapine and clozapine inhibit both dopamine D2 auto-receptors and serotonin 2A receptors, thus relieving positive Parkinsonism symptoms as well as negative schizophrenia symptoms [62]. Quetiapine and clozapine both share structural similarities with tricyclic antidepressants and particular D2 pharmacodynamics [63]. As such, quetiapine and clozapine have both been used in the treatment of psychosis in PD, unlike other antipsychotics (see below) [64].

### 2.3. Contraindicated Drugs in PD Therapy

Top associations also include haloperidol (4%) and sulpiride (3%). However, unlike quetiapine and clozapine, haloperidol and sulpiride are contraindicated in PD, due to motor symptom exacerbations and extrapyramidal effects [65]. This is because haloperidol and sulpiride block postsynaptic dopamine D2 receptors [66].

Top associations also include methyldopa (6%), an α2-adrenergic agonist used in the treatment of hypertension. Methyldopa can potentially produce Parkinsonism, and it is contraindicated in PD [67]. This is because methyldopa decreases dopamine levels [68].

### 2.4. Diagnostic Agents

Top associations also include diagnostic agents used in PD. For example, N-(3-fluoropropyl)-2β-carboxymethoxy-3β-(4-iodophenyl) nortropane (3-fluoropropyl, 60%), better known as FP-CIT, and radiolabeled with 18F, is used as a diagnostic marker of PD with positron emission tomography/computed tomography (PET/CT) [69]. Likewise, 2β-carboxymethoxy-3β-[4-iodophenyl] tropane, better known as beta-CIT (43%), radiolabeled with 123I, is also used as a diagnostic marker of PD [70]. These diagnostic agents are safe cocaine analogs, with a high affinity for dopamine transporters, and unlike long-term exposure to cocaine, they do not alter brain sensitivity to PD [71].

### 2.5. Biomarkers

Top associations also include PD biomarkers, such as 3-methoxytyrosine (48%) and homovanillic acid (12%). 3-methoxytyrosine (48%), better known as 3-O-methyldopa, is the main metabolite of L-dopa. 3-methoxytyrosine is a biomarker of PD, and it has been used to identify the response to L-dopa treatment [72]. Homovanillic acid (12%) is a downstream metabolite of dopamine, and it has also been used as a diagnostic marker of PD [73]. Such metabolites are considered good diagnostic biomarkers of PD, and cerebrospinal fluid (CSF) levels are decreased in untreated patients, increase after levodopa therapy, and correlate well with motor impairment.

### 2.6. Inducers of PD in Animal Models

Top associations include molecular compounds used in the chemical induction of PD in animal models, such as hydroxydopamine (40%), N-Methyl-4-phenyl-1,2,3,6-tetrahydropyridine (78%), tetrahydropyridine (77%), and probenecid (4%), as well as several neurotoxic quinolines like salsolinol (32%), 1,2,3,4-tetrahydroisoquinoline (16%), and quinolinic acid (4%).

6-hydroxydopamine (40%) is a synthetic neurotoxic compound derived from dopamine. 6-hydroxydopamine, also known as oxidopamine, has been used to induce PD in animal models [74]. 6-hydroxydopamine does not cross the BBB, and it is therefore injected directly into the substantia nigra. Inside neurons, 6-hydroxydopamine is further oxidized by MAO and converted into toxic products, like hydrogen peroxide, catecholamine quinones, and reactive oxygen species (ROS) and reactive nitrogen species (RNS) [75].

N-Methyl-4-phenyl-1,2,3,6-tetrahydropyridine (78%), better known as MPTP, and tetrahydropyridine (77%) are synthetic neurotoxins, and both are used to induce PD in animal models [76]. Notably, MPTP in combination with probenecid (4%) induces persistent depletion of dopamine in the striatum of a mouse model of PD [77]. Once injected, MPTP traverses the BBB and localizes in the substantia nigra, where it is further oxidized by MAOs to generate hydrogen peroxide and ROS [78].

Quinolinic acid (4%) is an endogenous tryptophan metabolite at the kynurenine level, and, unlike kynurenic acid, it is not neuroprotective [79]. Quinolinic acid does not cross the BBB, and following intranigral injection, it induces N-methyl-d-aspartate (NMDA) receptor and ROS- and RNS-dependent neurodegeneration in the striatum and hippocampus [80]. Quinolinic acid is also a precursor of nicotinamide adenine dinucleotide (NAD).

Another quinoline, 1,2,3,4-tetrahydroisoquinoline (16%), also known as TIQ, is also naturally present in the brain, where it and its alkylated derivatives are believed to contribute to PD. In contrast, certain TIQ derivatives, such as 1-methyl-TIQ has neuroprotective properties, while 1-Me-N-propargyl-TIQ inhibits the deleterious effects of MPTP [81]. TIQ derivatives are also found in various plants, such as tubocurarine from the South American liana (*Chondrodendron tomentosum*), and lotusine from the Formosan lotus (*Nelumbo nucifera*) [82].

Salsolinol (32%) is a neurotoxic isoquinoline that induces apoptosis of dopaminergic neurons due to its structural similarity to MPTP [83]. Salsolinol has been detected in the CSF of PD patients [84], and it is present in various edible plants, for example, in dried bananas and cocoa powder [85]. Notably, salsolinol neurotoxicity is attenuated by oral supplementation of neuroprotective acteosides [86]. Acteoside is a phenylethanoid glycoside and polyphenolic compound, which is abundant in olives (*Olea europaea* L.), tea olive flowers (*Osmanthus fragrans*), and Asiatic witchweed (*Striga asiatica*) [86].

Top associations include rotenone (25%), an isoflavone compound that naturally occurs in the jicama vine plant (*Pachyrhizus erosus*), as well as many Fabaceae plants. It has broad-spectrum insecticide and pesticide activity, and it is also toxic to fish. Rotenone does not cross the BBB, and following intracerebral injection, it progressively reproduces features of clinical PD, as well as modeling interactions of the gene–environment [87]. The dopaminergic cell loss induced by these neurotoxins leads to the development of Parkinsonian symptoms such as bradykinesia and muscle rigidity.

Finally, top associations also include the neurotoxic amino acid β-Methylamino-L-alanine (29%), also known as BMAA. BMAA was first separated from the seeds of queen sago (*Cycas circinalis*), and has been deemed a risk factor for PD [88]. BMAA induces neurotoxicity through multiple mechanisms, such as NMDA receptor activation [89]. Furthermore, BMAA, which is a non-proteinogenic amino acid, has been known to misincorporate into human proteins, instead of serine, leading to protein misfolding and aggregation [90]. Notably, BMAA has been detected in different tissue samples of organisms at various trophic levels in the Guam ecosystem, ranging from cyanobacteria, through cycad tissues and products, to flying foxes, and brain tissues of the local population [91]. Evidence for BMAA-triggered neurodegeneration is strongest among the Chamorro people of Guam, where chronic exposure to BMAA through consumption appears to induce Parkinsonism. As such, BMAA is a risk factor for PD.

### 2.7. Endogenous Cofactors

Top associations with PD also include potential therapeutic compounds, such as the endogenous cofactors coenzyme Q10 (4%) and tetrahydrobiopterin (5%). Coenzyme Q10 (4%), also known as ubiquinone, is an antioxidant that prevents the loss of dopaminergic neurons [92]. Coenzyme Q10 is a fat-soluble vitamin K-like cofactor that is essential for the respiratory chain in mitochondria, and PD patients suffer from low levels in their cerebellar cortex [93]. Coenzyme Q10 can be obtained from dietary sources, such as meat, fish, seed oils, and vegetables, as well as dietary supplements. Tetrahydrobiopterin (5%), also known as sapropterin and BH4, is a cofactor of tyrosine hydroxylase, the rate-limiting step of dopamine production. Tetrahydrobiopterin is also a cofactor of many other enzymes, such as phenylalanine hydroxylase, tryptophan hydroxylase, aromatic amino acid decarboxylases, alkylglycerol monooxygenase, and nitric oxide synthases. Although tetrahydrobiopterin administered orally does not improve the acute prognosis of PD patients [94], it does confer protection against MPTP-induced PD in a mouse model [95].

## 3. Discussion

In this study, we have explored the top molecules associated with PD, using their PubMed co-citation. The molecules have been grouped according to function, and they include FDA-approved drugs, disease inducers, and neuroprotective compounds. Approved drugs include L-dopa (49%) and carbidopa (63%). Adjunctive therapy includes droxidopa (33%) and trihexyphenidyl (28%). Diagnostic agents include beta-CIT (43%). Biomarkers include homovanillic acid (13%). PD inducers include 6-hydroxydopamine (40%) and MPTP (78%). Endogenous cofactors include tetrahydrobiopterin (4%) and ConenzymeQ10 (4%). Table 1 classifies the top molecules into approved drugs, adjunctive therapy, contraindicated drugs, diagnostic markers, endogenous cofactors, and inducers of PD.

While Parkinson’s disease (PD) is often diagnosed easily, termed a “waiting room diagnosis,” autopsy studies have revealed that 20% of patients initially diagnosed with PD have a different diagnosis upon autopsy [96]. The most common mimickers are Parkinsonian syndromes, which are other neurodegenerative disorders sharing some features with PD. These disorders have distinct clinical signs not typically seen in PD, known as “red flags,” and often show little or no response to PD treatments. While PD etiology is not completely understood, some of the risk factors are thought to be inherited, involving single or multiple genes. Monogenic forms account for less than 10% of all PD patients, and harbor mutations in autosomal-dominant genes, SNCA, LRRK2, and VPS35, or in autosomal recessive genes PINK1, DJ-1, and Parkin [97]. Polygenic forms harbor 26 PD risk loci; however, these show only moderate effects on PD risk. Thus, most PD cases are either misdiagnosed or simply due to external agents such as infectious diseases, including viruses [98] and bacteria [99], xenobiotics, including natural toxins and synthetic drugs [100], and deficiencies of vitamins and minerals. To prevent misdiagnosis, PD patients must undergo rigorous diagnosis before treatment options are considered.

### 3.1. Conditionally Essential Nutrients

**Figure 2:**
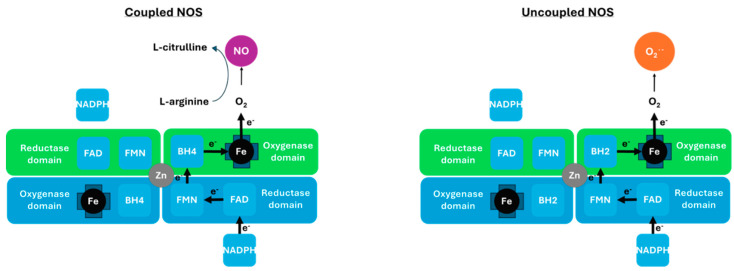
Nitric oxide synthase (NOS) coupling with biopterin. Nitric oxide synthase (NOS) functions as a homodimer, with each monomer having a reductase and an oxygenase domain. In the coupled state, electrons flow from nicotinamide adenine dinucleotide phosphate (NADPH) to flavin adenine dinucleotide (FAD) and flavin mononucleotide (FMN) in the reductase domain of one monomer to the iron (Fe) in the oxygenase domain of the other monomer. Tetrahydrobiopterin (BH4) binds near the iron active site, increasing L-arginine affinity to NOS, producing nitric oxide (NO) and L-citrulline. In the uncoupled state, with dihydrobiopterin (BH2) present, L-arginine affinity is lowered, and electron transfer and O_2_ reduction are uncoupled from L-arginine oxidation, generating superoxide (O_2_^•−^). Note that Coenzyme Q10 can quench superoxides and chelate excess iron.

Conditionally essential nutrients are biological compounds that are normally produced by the organism in sufficient amounts to meet its physiological needs. However, in disorders like PD and in other physiologically stressful conditions, their biosynthesis may be inadequate. Here, we hypothesize that the endogenous cofactors, tetrahydrobiopterin (BH4) and Coenzyme Q10 (CoQ10), are conditionally essential nutrients in PD patients. The relevance of small molecules to PD pathophysiology is further supported by recent work highlighting their role in modulating α-synuclein aggregation, oxidative stress, and neurodegeneration [101]. Our hypothesis is a secondary interpretation emerging from the association patterns and may be oversimplified. However, this hypothesis coincides with the finding that BH4 deficiency has severe consequences, as it is an essential cofactor for dopamine and serotonin biosynthesis, as well as all isoforms of nitric oxide synthase (NOS). As such, BH4 deficiency reduces the levels of dopamine, serotonin, and nitric oxide (NO) (Figure 2). Limited BH4 can also lead to NOS uncoupling with BH2, and production of superoxides instead of NO. While BH4 administered orally does not improve the symptoms of PD patients [94], it does protect against MPTP-induced neurotoxicity in a PD mouse model [95]. Clinical investigations of BH4 have yielded mixed results. Early phase II trials have shown symptomatic improvement, but subsequent phase III trials have failed to confirm efficacy, likely due to limited bioavailability and poor BBB penetration [92]. BH4’s failure to improve PD symptoms may also be due to inadvertent degradation by the microbiome, oxidation into dihydrobiopterin (BH2), and restriction at cofactor exchange [94]. In fact, tetrahydrobiopterin (BH4) may require intravenous injections, or perhaps even an intranasal formulation to reach its target.

Without antioxidants like CoQ10, to quench superoxides in Figure 2, critical enzymes, including those in the mitochondrial electron transport chain, can suffer irreversible damage. This cascade of events is hypothesized to occur in PD [102], and agrees with the findings that antioxidants found in licorice, curcumin, and cocoa, and vitamin E reduce the risk of PD through quenching of superoxides. Furthermore, neurotoxic quinolones and isoquinolones can interfere with BH4 biosynthesis, prevent enzyme catalysis, and uncouple NOS [103]. Dietary quinolones can therefore inhibit dopamine, serotonin, and NO production, as well as increase superoxide production. This could explain how inhibition of neuronal NOS prevents MPTP-induced Parkinsonism [104]. Notably, quinolone interference with BH4 biosynthesis could depend on its structure, and while TIQ could uncouple NOS, 1-Me-N-propargyl-TIQ would not [81]. Finally, BH4 supplementation inhibits ferroptosis [105], Coenzyme Q10 can manage iron levels through chelation [106], and together they can prevent the accumulation of excess iron. As such, these compounds represent a mechanistic bridge between metabolism, oxidative stress, and dopaminergic neurodegeneration. Their “conditional essentiality” under stress makes them a key target for PD management.

Future BH4 and CoQ10 studies should explore alternative delivery routes (e.g., intranasal, intravenous) to enhance bioavailability, different formulations (e.g., precursors, liposomal nanocarriers) to increase stability, and combination therapies with tyrosine hydroxylase substrates (i.e., tyrosine), as well as NOS substrates (e.g., L-arginine), to maximize effect on PD patients.

### 3.2. Limitations and Potential Pitfalls

The association reported herein is derived from large-scale PubMed co-citation analysis and, as such, is subject to methodological limitations inherent to bibliometric and text-mining approaches. First, co-citation does not imply causation, nor does it distinguish between positive, negative, mechanistic, or incidental relationships. A molecule may be cited in the context of therapeutic benefit, toxicity, diagnostic relevance, or merely as background information.

Second, citation frequency is influenced by several external factors, including historical research trends, funding priorities, and molecule ubiquity. Highly ubiquitous molecules are extensively cited across diverse biological contexts, which can inflate absolute citation counts without reflecting disease specificity. Conversely, molecules that are more narrowly studied in the context of PD may show fewer absolute citations, yet demonstrate a stronger relative association with PD. To partially mitigate this bias, we employed normalized association measures, defined as the ratio between PD-molecule co-citations and total molecule citations. This normalization aims to highlight preferential associations rather than overall popularity. Nevertheless, normalization itself introduces limitations, as it may underrepresent broadly studied molecules with genuine relevance to PD, and it remains sensitive to publication practices and temporal trends.

Third, bibliometric results are database-dependent. PubMed and Google Scholar differ substantially in scope, indexing criteria, inclusion of non-peer-reviewed material, and citation aggregation methods. Therefore, absolute citation numbers may vary widely between databases and should not be interpreted as interchangeable. In this study, PubMed was chosen for its curated biomedical focus and consistent indexing, but we acknowledge that results obtained from alternative databases may differ.

Fourth, associations are time-dependent. Molecules that were intensively studied during specific historical periods may appear prominently despite reduced contemporary relevance, while emerging compounds may be underrepresented. To reduce random co-occurrence and background noise, we restricted analyses to title and abstract fields and applied a minimum co-citation threshold of 100, assuming a conservative signal-to-noise criterion. While this improves robustness, it does not eliminate all sources of bias.

Finally, this review is not intended to provide an exhaustive inventory or definitive ranking of all molecules related to PD. Rather, it aims to identify consensus patterns that emerge from large-scale literature mining and to generate hypotheses regarding conditionally essential cofactors and metabolic vulnerabilities in PD. The findings should therefore be interpreted as hypothesis-generating rather than conclusive and should be integrated with experimental, clinical, and mechanistic evidence.

## 4. Materials and Methods

List of Compounds: To prepare a list of molecular compounds found in food and drugs, we downloaded a comprehensive list of 217,776 molecules identified in the Human Metabolome Database (HMDB 5.0) [35]. The full HMDB names of molecular compounds were used as the ‘molecular term’ in our PubMed queries, and were not abbreviated.

PubMed Count: To mine for foods and drugs associated with PD, we queried PubMed for molecule terms using a Python script (version 3.12 vas) with the Requests library to download and the BeautifulSoup library to parse HTML files. First, the program counted the number of PubMed co-citations with PD and the molecule term (e.g., “Parkinson” AND “dopamine”) and for the molecule alone (e.g., “dopamine”). Then, to normalize the association, the program divided the number of co-citations by the number of citations of the molecule alone. Normalization reduced the bias introduced by overly abundant or rare terms and minimized the influence of publication trends rather than true biological associations. Moreover, to ensure statistical robustness, the program restricted queries to title/abstract fields and required at least 100 co-citations. Finally, the normalized association was multiplied by 100 to obtain a percentage value. The normalized PubMed association corresponded to the generalized formula:

$${Normalized\;Association}_{Molecule+PD}\;=\;\frac{\left[\;C{itations}_{Molecule+PD}\;\right]}{\left[{Citations}_{Molecule}\right]}\;\times \;100$$

To obtain accurate results for multi-word terms, parentheses with double quotes were used in all our searches. Our program performed ~1600 searches per hour and printed the normalized associations to a comma-separated value text file, which could be imported into Excel for easy sorting.

## 5. Conclusions

This review uses a large-scale PubMed mining of 217,776 HMDB-listed compounds to identify consensus food and drug molecules in Parkinson’s disease (PD). Our analysis successfully captures well-established therapeutic agents, adjunctive treatments, contraindicated drugs, diagnostic markers, biomarkers, and inducers of Parkinsonism. Moreover, our study highlights endogenous cofactors, tetrahydrobiopterin (BH4) and Coenzyme Q10 (CoQ10), responsible for proper tyrosine hydroxylase and NOS catalysis that could become essential in PD. While our results highlight rational directions of investigation in PD, translation into effective interventions requires optimizing formulation to improve CNS delivery.

## Figures and Tables

**Table 1:** Classification of top associations.

Role in Parkinson’s Disease	Molecule
Drug	L-Dopa (49%), Carbidopa (63%), Benserazide (50%), Entacapone (74%), Tolcapone (56%), Rasagiline (76%), Selegiline (46%), Pargyline (4%), Ropinirole (61%), Rotigotine (61%), Pramipexole (56%), Pergolide (47%), Lisuride (27%), Apomorphine (19%), Cabergoline (16%), Bromocriptine (12%), Zonisamide (9%)
Adjunctive therapy	Droxidopa (33%), Trihexyphenidyl (28%), Biperiden (17%), Amantadine (24%), Memantine (7%), Rivastigmine (13%) Donepezil (6%), Galantamine (4%), Domperidone (6%), Clonazepam (4%), Tetrabenazine (16%), Mazindol (13%), Quetiapine (6%), Clozapine (4%)
Contraindicated drug	Haloperidol (4%), Sulpiride (3%), Methyldopa (6%)
Diagnostic agent	FP-CIT (60%), Beta-CIT (43%),
Biomarker	3-Methoxytyrosine (48%), Homovanillic Acid (12%)
Endogenous cofactors	Coenzyme Q10 (4%), * Tetrahydrobiopterin (4%),
Inducer	6-Hydroxydopamine (40%), MPTP + Probenecid (78% + 4%), Tetrahydropyridine (77%), * TIQ (16%), * Salsolinol (32%), * Quinolinic Acid (4%), Rotenone (25%), BMAA (29%)

* Quinoline skeleton.

## Data Availability

No new data were created or analyzed in this study.

## Abbreviations

The following abbreviations are used in this manuscript:

AADC—aromatic L-amino acid decarboxylase; BH2—dihydrobiopterin; BH4—tetrahydrobiopterin; BMI—body mass index; CNS—central nervous system; BMAA—β-Methylamino-L-alanine; COMT—catechol-O-methyltransferase; FAD—flavin adenine dinucleotide; FMN—flavin mononucleotide; HMDB—Human Metabolome Database; NAD—nicotinamide adenine dinucleotide; NMDA—N-methyl-d-aspartate; MAO—monoamine oxidase; MPTP—N-Methyl-4-phenyl-1,2,3,6-tetrahydropyridine; PD—Parkinson’s disease; PUFA—polyunsaturated fatty acid; ROS—reactive oxygen species; RNS—reactive nitrogen species.
